# Growth Hormone Deficiency, Short Stature, and Juvenile Rheumatoid Arthritis in a Patient with Autoimmune Polyglandular Syndrome Type 1: Case Report and Brief Review of the Literature

**DOI:** 10.5402/2011/462759

**Published:** 2011-05-04

**Authors:** Teresa Pun, Vikram Chandurkar

**Affiliations:** ^1^Internal Medicine, Memorial university of Newfoundland, Canada A1B 3V6; ^2^Division of Endocrinology, Health Sciences Center, Faculty of Medicine, Memorial University of Newfoundland, 300 Prince Philip Drive, St. John's, NL, Canada A1B 3V6

## Abstract

Autoimmune polyglandular syndromes (APSs) include a cluster of autoimmune and nonautoimmune conditions which have been classified into subtypes. APSs type 1 is characterized by at least two of the following: chronic mucocutaneous candidiasis, chronic hypoparathyroidism, and autoimmune Addison's disease (AD). We report the chronological history of a female patient who presented with features most consistent with APS type 1, along with growth hormone deficiency and juvenile rheumatoid arthritis (JRA). In terms of her autoimmune diagnoses, she first presented with JRA at three years of age, then hypocalcemia and hypoparathyroidism at five years of age, type 1 diabetes (DM 1) at age eleven years, adrenal insufficiency at age fourteen years, recurrent mucocutaneous candidiasis as a teenager, growth hormone deficiency at age fourteen years leading to significant short stature, primary amenorrhoea, and hypogonadism, and finally alopecia at age twenty-six years. In addition to this, she has suffered other nonautoimmune medical problems including a Tetralogy of Fallot with a surgical repair at age six years. On review of the medical literature, we found no other previously reported case with this unique combination of medical problems.

## 1. Introduction

Autoimmune polyglandular syndromes (APSs) include a cluster of autoimmune and nonautoimmune conditions which have been classified into four types by Neufeld et al. in 1980 [1, 2]. APS type 1 is characterized by at least two of the following: chronic candidasis, chronic hypoparathyroidism, and autoimmune Addison's disease (AD). APS type 2 is defined by autoimmune AD (must be present) and autoimmune thyroid disease and/or type 1 diabetes mellitus. APS type 3 involves thyroid autoimmune disease and other autoimmune diseases (excluding AD, hypoparathyroidism and chronic candidiasis). Lastly, APS type 4 describes the coexistence of two or more organ-specific autoimmune diseases and does not fulfill the criteria of the previous types of APS [1, 2]. 

Other conditions associated with APS 1 include autoimmune thyroid disease, type 1 diabetes (DM 1), hypogonadism, alopecia, vitiligo, keratopathy, autoimmune hepatitis, pernicious anemia, and chronic gastritis [3]. 

APS 1 has been linked to a defect of the autoimmune regulator (AIRE) gene, located on chromosome 21q22.3. This gene, when defective, downregulates the transcription of several tissue-restricted antigens (TRA), resulting in the decreased clonal deletion of autoreactive T cells [4].

We report the chronological history of a female patient who presented with features most consistent with APS type 1, along with a variety of other autoimmune and nonautoimmune conditions from birth.

## 2. Case Report

On maternal obstetrical history, the patient's mother was G13P10A3. She was forty-one years old at the time of birth and prenatal complications included per vaginal bleeding at five months of gestation. The patient was born by Caesarean section at thirty-four weeks gestation by dates. APGAR scores were 6, 8, and 10 at 1, 5, and 10 minutes, respectively. Her birth weight was 1665 grams. Since birth, her height and weight have been consistently beneath the fifth percentile. A karyotype was negative for Turner's syndrome, and thyroid function testing was normal. 

With respect to her development, the patient demonstrated significant motor retardation; she was unable to walk independently until the age of three. There was some discussion of mild mental retardation initially, but she completed grade eleven when she was eighteen years old. As a child and young woman, she has occasionally been described as elfin and fawn-like.

At three months of age, the patient was diagnosed with a ventricular septal defect (VSD) with a left- to right- shunt. She was subsequently diagnosed with a moderate- to- severe Tetralogy of Fallot and a double-chamber right ventricle by cardiac catheterization. This was surgically repaired when the patient was six years old. 

At three years of age, she was diagnosed with juvenile rheumatoid arthritis (JRA), which was treated with nonsteroidal anti-inflammatory drugs (NSAIDs) initially and later, with intermittent courses of prednisone. Her ESR was elevated at 31 mm/HR. Her antistreptolysin O titer (ASOT), rheumatoid factor (RF) and antinuclear antibody (ANA) were negative. Her symptoms decreased in severity over time although she remains with a 2 cm leg length discrepancy in her left leg. She has been involved with physiotherapy since childhood, and they have often remarked on her hypermobile limbs.

The patient suffered from dental caries from an early age. She had thirteen extractions, and upon examination, none of the teeth appeared hypoplastic. She had middle ear issues as well, requiring bilateral tympanostomy tubes and a left mastoidectomy for treatment of cholesteatoma. At the age of five years, she was diagnosed with conductive hearing loss, requiring the use of hearing aids.

At five years of age, she presented with seizures, which were initially attributed to hypocalcemia due to hypoparathyroidism. She was started on vitamin D replacement with cacitriol. Further investigations included an EEG which demonstrated diffuse encephalopathy and slowing of cortical rhythms over bilateral hemispheres. An X-ray brain scan was normal (AP, PA, lateral views × 2). The patient was diagnosed with a seizure disorder and started on Phenobarbital. She was subsequently diagnosed with megaloblastic anemia, presumably secondary to Phenobarbital therapy. She was started on folic acid and vitamin B12 supplementation.

Shortly afterwards, the patient was diagnosed with irritable bowel syndrome and has been on ongoing treatment throughout her adulthood. She continues to have issues with constipation.

At eleven years of age, she was diagnosed with DM 1. This was treated with insulin, and she is currently on a regime of regular insulin with meals and NPH insulin at bedtime. 

After her diagnosis of diabetes, the patient had some symptoms consistent with adrenal insufficiency; she had a workup which yielded a normal basal cortisol level and an appropriate, response following ACTH stimulation. Three years later, both her basal cortisol (100 nmol/L, range 120 to 600 nmol/L) and ACTH levels were abnormally low, and the response to an ACTH stimulation test was negligible. Cortisol levels were 160 nmol/L and 200 nmol/L. There were concerns regarding primary ACTH deficiency versus prolonged suppression of ACTH production due to previous prednisone therapy. She was initially treated with prednisone and florinef in the morning. This was subsequently changed to prednisone on a twice daily basis. On prednisone alone, she had no postural hypotension, decreased episodes of hypoglycemia overnight, and a normal potassium. Subsequent testing, when the patient was thirty-six years old, demonstrated a detectable ACTH level, suggesting a possible diagnosis of primary adrenal insufficiency. Ultrasound of the adrenal glands was normal.

The patient had recurrent episodes of chronic mucocutaneous candidiasis and angular stomatitis in childhood and continued to have episodes intermittently. Skin testing revealed nonreactivity to multiple fungi, suggesting a deficit in cell-mediated immunity. She was treated with several courses of fluconazole with minimal success. Serum gamma globulin levels were normal for IgG, IgA, and IgM. She has also had several admissions for pneumonia.

She was investigated for her short stature at the age of fourteen years, with a bone age of eight years and ten months. Sleeping growth hormone levels were normal at 32.5 micrograms/L (0.0 to 7.7 micrograms/L), but the response of growth hormone to arginine stimulation was significantly depressed. Growth hormone levels were 5.0 micrograms/L, 0.8 micrograms/L, and 1.4 micrograms/L at time zero, thirty minutes, and sixty minutes after arginine administration. Her growth hormone level was 0.5 micrograms/L after twenty minutes of exercise. She was treated with a course of Fluoxymesterone (Halotestin), and when she was sixteen years old, she was treated with a trial of growth hormone injections but with limited success. At the onset of therapy, her bone age was eight years and six months. When the patient was nineteen years old, her height was 124.5 cm, and her bone age was eleven years. Her final height was 130 cm. TSH was normal at 1.68 mU/L (0.3 to 3.8 mU/L). There is no family history of short stature or failure to thrive.

The patient suffered from primary amenorrhea with no development of secondary sexual characteristics. FSH and LH levels were high at 95 U/L and 46 U/L, respectively, and both responded well to LHRH and TRH stimulation. FSH and LH were 135 U/L and 160 U/L at time zero and greater than 145 U/L and 137 U/L, respectively, at fifty minutes. These findings were consistent with primary ovarian failure. Treatment with hormone therapy was delayed in favour of initial treatment with growth hormone. She was subsequently started on Estrogen supplement (Premarin) and Medroxyprogesterone acetate (Provera). The patient chose to discontinue treatment herself when the results of the Women's Health Initiative (WHI) were made public, but re-started when she was diagnosed with marked osteoporosis shortly afterwards.

At the age of nineteen years, the patient was diagnosed with bilateral cataracts (postcapsular, nuclear, and cortical). There was no evidence of diabetic retinopathy.

At the age of twenty-six years, she presented with alopecia totalis. She has sparing of the eyelashes. She also presented with dysphagia at this time and was diagnosed with esophageal webs. She underwent several dilatations for esophageal stenosis. At this time, she was also diagnosed with pernicious anemia and started on vitamin B12 supplementation. 

Two years later, at twenty-eight years of age, routine screening documented elevated creatinine with an elevated urine microalbumin at 134 mg/L. She was started on ramipril for diabetic nephropathy. An ultrasound of the kidneys showed evidence of nephrocalcinosis. The patient's kidney function continued to deteriorate, and at the age of thirty-five years, she was started on hemodialysis, eventually switching to peritoneal dialysis. Her kidney function started improving after a short course of dialysis, and she was able to discontinue it.

She was seen in the endocrinology clinic at this time, and her assessment is summarized here.

Her current medications include:

1. Calcitriol (Rocaltrol) 0.25 micrograms once daily.
2. Prednisone 5 mg once daily in the morning.
3. Fludrocortisone (Florinef) 0.1 mg once daily.
4. 1 unit of Novolin Toronto Insulin mixed with 7 units of NPH in a syringe which she takes between 7 a.m. and 10 a.m. and the same dose, 1 unit of Toronto with 7 units of NPH insulin, at 4:30 p.m..
5. Premarin 0.625 mg from day 1 to day 25 of each month.
6. Provera 2.5 mg on day 15 to day 25 of each month.
7. Vitamin B12.
8. Diflucan as needed.
9. Nystatin oral suspension as needed.
10. Colace prn.

### 2.1. Allergies

The patient has experienced allergic reactions to Ceftin, Ciprofloxacin, and Bactrium. She presented with a rash to all of these agents.

### 2.2. Family History

The patient has six brothers and three sisters. One brother has adrenal insufficiency, hypoparathyroidism, alopecia, and anemia, but he does not have diabetes. Two sisters and two brothers have rheumatoid arthritis. One sibling died at the age of three and a half years. There is no history of consanguinity. Her father died at the age of seventy-one years due to metastatic cancer with an unknown primary. Her mother died at the age of seventy-one years of bowel carcinoma; she also had hypertension. Her maternal grandmother had Type 1 DM.

### 2.3. Social History

The patient is a life-time nonsmoker and does not drink any alcohol. She is not working, and she currently lives with her brother.

### 2.4. Review of Systems

 The patient has gained weight recently since her most recent esophageal web surgery. Her bowel movements have been regular. She denies any fatigue, tiredness, or dizzy spells.

### 2.5. Physical Examination

Height was 129.8 cm. Weight was 29 kg. Calculated body mass index (BMI) is 17.2. Blood pressure was 100/60 mmHg. On general examination, the patient is a petite female of short stature in no apparent distress. She has alopecia totalis and wears a wig. There is no lid lag or proptosis. On examination of her thyroid, each lobe measured about 2 cm in vertical dimension. There was some prominence in the right lobe in the central part, but there was no obvious nodule. On cardiovascular examination, the rhythm was regular. Lungs were clear to auscultation. There was no edema in the lower extremities. Sensation to the 10 g monofilament was largely intact. There was one patchy area over the right heel where the monofilament felt different when compared to the rest of the sole. Dorsalis pedis pulses were 2 + bilaterally. She has been injecting her insulin mostly in her arms recently. There were some small bruises in these areas. The patient does have evidence of past rheumatoid arthritis involving her hands. She did have a peritoneal dialysis catheter placed in the right side of her abdomen.

### 2.6. Recent Investigations

She underwent a combined pituitary stimulation test in the clinical investigation unit (CIU); this included an insulin hypoglycemia component. Glucose at baseline was 15.5 mmol/L. She received 6 units of insulin, and her glucose dropped to 2.5 mmol/L at 60 minutes and 1.0 mmol/L at 90 minutes. Cortisol did not show any response. At baseline, it was 17 mmol/L, at 30 minutes it was 21 mmol/L, at 60 minutes 19 mmol/L, and at 90 minutes 18 mmol/L. ACTH levels were 8.0 pmol/L at baseline and 7.7 pmol/L at 60 minutes (normal range 0–10 pmol/L). On the GnRH test: FSH went from baseline of 11.4 U/L to 26.9 U/L at 90 minutes. LH went from 3.5 U/L at 0 minutes to 36.6 U/L at 90 minutes. TSH rose from 1.84 mU/L at 0 minutes to 4.08 mU/L at 60 minutes. Her most recent HbAIC was 7.0%. She did speak to a genetics counsellor but declined testing for the autoimmune regulator (AIRE) gene. Her recent laboratory investigations are summarized in Table 2.

## 3. Discussion

On review of the medical literature, we found no other previously reported case with this unique combination of medical problems. Our patient had evidence of selective pituitary dysfunction with growth hormone deficiency despite a normally functioning thyroid axis. One other case report discusses the coincidence of growth hormone deficiency and APS 1. Franzese et al. describe a female patient who developed growth hormone insufficiency with delayed bone age at the age of eight years. She also presented with hypothyroidism and gonadal failure. She was treated with recombinant human growth hormone when she was ten years old and was able to achieve her target height on this therapy [5]. In her case, the growth hormone insufficiency was attributed to a partially empty sella found on computed tomography scanning of the head [5]; however, it does not explain why her deficit was manifested so late in her childhood.

The patient in our case report likely developed difficulties with growth hormone insufficiency early in infancy, as she has always been and remains beneath the fifth percentile for height and weight. Her poor response to treatment with growth hormone may be due to her prolonged duration of growth hormone insufficiency. In another case report by Ward et al. [6], autoimmune hypophysitis was thought to have caused growth hormone deficiency in a patient with Autoimmune polyendocrinopathy-candidiasis-ectodermal dystrophy (APECED). This diagnosis was supported by an appearance of ring enhancement on MRI.

Our patient also had high gonadotropin levels, indicating that her pituitary gonadotrophs were also intact. The etiology of hypogonadism in this patient is likely autoimmune. Studies have shown that ovarian failure in APS type 1 patients is associated with autoantibodies to several reproductive tissues, including cells in the adrenal cortex (which secrete steroid hormone), Graffian follicles, and corpus luteum [7].

Though the patient's initial workup for adrenal insufficiency was not clearly diagnostic of primary adrenal insufficiency, subsequent testing did reveal detectable ACTH levels. The diagnosis was likely confounded by early treatment with prednisone for JRA, leading to the suppression of ACTH production.

At this point, we cannot rule out an autoimmune origin for our patient's pituitary deficits, such as a limited lymphocytic hypophysitis. This may partly explain the growth hormone insufficiency, in the midst of normal thyroid function.

More unique findings in our case include the presence of Tetralogy of Fallot and JRA in a patient with APS 1. To our knowledge, this is the first case report that documents the coexistence of both of these conditions in APS 1. There is a case, however, describing the presence of an atrial septral defect (ASD) in an APS 2 patient [8]. Congenital heart defects, including septal defects, have been linked to many genetic syndromes, such as Down's syndrome [8]. Interestingly, the genetics of DiGeorge syndrome, a deletion in chromosome 22q11, may be partly responsible for the different Tetralogy of Fallot phenotypes exhibited in this syndrome [9]. DiGeorge syndrome also manifests with an absent thymus and resulting T cell defects and one of the hypotheses behind APS type 1 involves isolated T cell defects [10]. The AIRE gene, which is defective in APS type 1, is mapped to a different chromosome—chromosome 21q22.3 [10]. The increased incidence of autoimmune diseases in Down syndrome patients is interesting in light of the location of the AIRE gene on chromosome 21. The addition of an extra AIRE gene, functional or defective, may have implications in the presence or severity of the autoimmune phenotype in a particular individual.

We could find only 2 previous case reports of JRA in an APS type 1 patient [11]. There is a report of an APS type II patient with rheumatoid arthritis [12]. In our case, the patient was RF negative.

The 2 largest case series reported with APS type 1 are from Finland by the same author possibly including some common patients: one with 68 cases reported in 1990 did not mention growth hormone deficiency, short stature, JRA, or Tetralogy of Fallot as a manifestation of APS 1 [13]. The other report of 91 cases mentions 5 cases of growth hormone deficiency but none with JRA [14].

## 4. Conclusion

This is the case of an APS type 1 patient presenting with several unusual, and atypical features including growth hormone deficiency, short stature, JRA and Tetralogy of Fallot. The inclusion of such cases in the medical literature allows researchers to link disparate diagnoses from a clinical viewpoint and to provide thought-provoking hypotheses to promote further exploration of the underlying mechanisms.

## Figures and Tables

**Table 1:** Chronologic development of manifestations of APS and other clinical features.

Diagnosis	Age at diagnosis
Low birth weight: 1665 gms	0
VSD	3 months
Juvenile rheumatoid arthritis	3 years
Hypoparathyroidism	5 years
Irritable bowel syndrome	6 years
Repair of Tetralogy of Fallot	6 years
Chronic mucocutaneous candidiasis	8 years^?^
Type 1 diabetes	11 years
Adrenal Insufficiency	12 years
Growth hormone deficiency	14 years
Ovarian Failure	15 years
Alopecia totalis	26 years
Pernicious anemia	26 years

^?^Date is uncertain from the medical record.

**Table 2:** Selected relevant laboratory parameters.

Laboratory parameter	Result	Normal range (units)
HbA1C	7.0	(4.0–6.0 gm%)
Parathyroid hormone	<3 ng/L	12–65 ng/L
ACTH	8.0 pmol/L	10–65 ng/L
TSH	0.82	0–10 pmol/L
LH	80.2	0.34–5.6 mIU/L
FSH	265.9	1.2–12.9 IU/L
Estradiol	<73	3.8–8.8 IU/L
